# CEBPB is associated with active tumor immune environment and favorable prognosis of metastatic skin cutaneous melanoma

**DOI:** 10.3389/fimmu.2022.991797

**Published:** 2022-10-24

**Authors:** Jingrun Yang, Yang Xu, Kuixia Xie, Ling Gao, Wenying Zhong, Xinhua Liu

**Affiliations:** ^1^ Department of General Surgery, The First Medical Center of Chinese People’s Liberation Army (PLA) General Hospital, Beijing, China; ^2^ Key Laboratory of Bio-inspired Materials and Interfacial Science, Technical Institute of Physics and Chemistry, Chinese Academy of Sciences, Beijing, China; ^3^ Department of Biochemistry and Molecular Biology, School of Basic Medical Sciences, Hangzhou Normal University, Hangzhou, China; ^4^ Department of Dermatology, The Affiliated Hospital of Hangzhou Normal University, Hangzhou, China; ^5^ Key Laboratory of Aging and Cancer Biology of Zhejiang Province, Hangzhou Normal University, Hangzhou, China

**Keywords:** CEBPb, SKCM, tumor immune microenvironment, single cell RNAseq, immune subtype

## Abstract

Metastatic skin cutaneous melanoma (SKCM) is a common malignancy that accounts for low morbidity but high mortality of skin cancer. SKCM is characterized by high lymphocytic infiltration, whereas the states of infiltrated cells are variable in patients leading to a heterogeneous prognosis and hindering appropriate clinical decisions. It is therefore urgent to identify markers associated with lymphocytic infiltration, cellular conditions, and the prognosis of SKCM. In this study, we report that CEBPB, a transcriptional factor, is mainly expressed in macrophages in metastatic SKCM and associated with an active tumor immune environment and a favorable prognosis through integrated analysis of single-cell and bulk RNA-seq datasets. High CEBPB expression is significantly associated with active inflammation and immune response pathways in both macrophages and bulk SKCM tumor tissues. A signature based on CEBPB-associated genes that are specifically expressed in macrophages could robustly and prognostically separate different metastatic SKCM patients. In addition, the associations between the metastatic SKCM tumor signature and microenvironment with respect to T-cell recruitment and state, inflammation response, angiogenesis, and so on were also determined. In conclusion, we present here the first report on CEBPB in tumor immune environment and prognosis regulation in metastatic SKCM and construct a reliable signature, which should provide a useful biomarker for stratification of the patient’s prognosis and therapeutic selection.

## Introduction

Skin cutaneous melanoma (SKCM), which occurs in the skin, is the most common type of melanoma. SKCM is one of the most aggressive and lethal malignancies, which accounts for only 3–5% of morbidity but almost 75% of mortality of skin cancer (1). Surgical resection followed by adjuvant chemotherapy or radiotherapy to control tumor spread represents the optimal therapeutic method for localized SKCM (2). Additionally, the development of targeted therapy and immunotherapy has significantly prolonged the overall survival (OS) of SKCM patients (3, 4). However, metastasis continues to pose a great threat to the prognosis of SKCM patients, and the probability of 5-year OS of metastatic SKCM is only approximately 19% (5). Systemic treatment, including chemotherapy and a combination of chemotherapy and biochemotherapy, has been extensively used in metastatic SKCM, but only low clinical benefits have been achieved and were often accompanied by great toxicity (6). A high level of lymphocytic infiltration in both primary lesions and metastatic sites enables SKCM to be an ideal candidate for immunotherapy, which aims to modulate the immune environment of tumor tissue, and a significant improvement and less toxicity compared to conventional chemotherapy are obtained (7). In contrast, great heterogeneities of response to immunotherapy exist in metastatic SKCM patients, largely due to the heterogeneous tumor microenvironment (TME) (8, 9). As a result, the identification of molecular biomarkers for metastatic SKCM stratification with different TME would be helpful for appropriate clinical decision-making.

The TME is a complex community composed of multiple cell types and interactions among them. Lymphocytes and myeloid cells could infiltrate the solid tumor tissue through tumor vasculature or other means, consisting of the immune part of the TME (9). Infiltration levels and states of cells are dynamically regulated by various factors. For example, the migration of T cells from the second lymphoid organ to the solid tumor tissue is regulated by some chemokines (10). High expression of PD-1, CTLA4 on the T cell surface, or PD-L1/L2 on the malignant cell surface could induce the exhaustion of T cells, resulting in the reduction and even the loss of their cytotoxicity on malignant cells (11). Cell–cell interactions *via* an autocrine or paracrine manner could also influence the states of infiltrated cells in the TME. Such malignant cells could induce the differentiation of fibroblasts into cancer-associated fibroblasts (CAFs) and promote tumor metastasis. Macrophage polarization to the M2 subtype induced by CAFs could in turn result in a suppressed immune environment mainly through manipulation of T-cell states (12). Additionally, it turned out that a growing number of molecules were involved in the regulation of the TME. For example, PTEN has been extensively studied solely in the epithelial context as a tumor suppressor, whereas its indispensable role in CAF regulation in the TME has recently been identified and approved for participation in cancer progression (13). CDR1, as a circular RNA, was found to be closely associated with the infiltration and state of immune cells in multiple cancers (14). However, the understanding of TME regulation in cancers, including metastatic SKCM, is far from complete, which represents an obstacle to the development of therapeutic methods and the determination of appropriate treatments.

CEBPB is a transcriptional factor (TF) that has three subtypes, namely, LAP-1, LAP-2, and LIP. LAP-1 and LAP-2 play important roles in transcriptional activation, while LIP is mainly involved in transcriptional repression (15). One of the most known functions of CEBPB is immune modulation in multiple inflammatory diseases, including sepsis (16, 17) and skin or fibrotic diseases (18–20). Furthermore, it has been demonstrated that CEBPB is the master regulator of macrophage differentiation, which could define the macrophage identity by marking the macrophage-specific chromatin regulatory elements for the following TF binding (21). The regulatory roles of CEBPB in the onset of cancer and the progression of its transcriptional regulatory function in malignant cells have also been uncovered (22, 23). A few studies have also reported potential associations between CEBPB and metastasis (24) or treatment response in melanoma (25). However, relationships between CEBPB and tumor immune landscape as well as the prognosis of metastatic SKCM have never been systemically reported.

In this study, we report that CEBPB is mainly expressed as macrophages in the TME of metastatic SKCM and is associated with an active immune environment. A signature based on CEBPB-regulated genes that are expressed specifically in macrophages could robustly separate prognostically and immunologically different metastatic SKCM patients.

## Materials and methods

### Datasets, normal fibroblast, and melanoma cell line

A total of three metastatic SKCM datasets were used for survival analysis in this study. The TCGA-SKCM cohort contains a total of 472 samples including one adjacent normal tissue sample, out of which 349 metastatic ones, including distant metastasis, regional lymph node metastasis, and regional skin metastasis, with complete prognosis information, were retained in this study. Another metastatic SKCM cohort was obtained from the study of Garraway et al. (26) and abbreviated as DFCI2015 hereafter, which contains 40 metastatic SKCM samples with transcriptome and complete survival information freely available. Notably, all 40 patients of the DFCI2015 cohort experimented with anti-CTLA4 treatment and the molecular data were obtained before the treatment. GSE59455 (27) from the Gene Expression Omnibus (GEO; https://www.ncbi.nlm.nih.gov/geo/) contains 141 SKCM samples including 39 primary and 102 metastatic ones, only those metastatic samples were retained for analysis, and all the samples were deceased at the end of the follow-up. Additionally, GSE46517 (28) contains 121 samples including 73 metastatic SKCM samples, 31 primary SKCM samples, nine nevus samples, and seven normal skin samples, and one normal epithelial melanocyte sample was also included in this study for CEBPB expression comparison. CEBPB mRNA level was compared between the 73 metastatic SKCM and the seven normal skin samples. Gene Expression Profiling Interactive Analysis (GEPIA; http://gepia.cancer-pku.cn/) online tool was also adopted to estimate the difference in CEBPB mRNA level by including the SKCM tumor samples from The Cancer Genome Atlas (TCGA) and normal skin samples from Genotype-Tissue Expression (GTEx; https://www.gtexportal.org/). A single-cell RNA-seq dataset GSE115978 (29), which contains gene expression profiles of 7,186 cells from 31 melanoma samples, was used for CEBPB cell-specific expression analysis.

Normal dermal fibroblast was obtained from a 67-year-old male keloid patient in a Chinese PLA hospital with signed consent obtained. The ethics committee of PLA General Hospital approved this study (Ethics Approval No. S2018-223-02). The details for fibroblast extraction and culture could be found in our previous study (30). Additionally, skin melanoma cell lines A375 and SK-MEL-2 were obtained from the RE-STEM cell bank (Jiangsu, China).

### Cell culture

A375 cells were cultured in a complete medium (DMEM:serum:double antibody = 100:10:1) and placed in a 5% CO_2_, 37°C incubator for culture. Subculture was carried out every 3 days, and seeds were grown on plates.

### Transfection

A total of 1 × 10^5^ cells were plated on a 6-well plate and transfected with 2 μg of either pcDNA3.1-3×Flag as mock control or CEBPB-pcDNA3.1-3×Flag. Transfection was done using 4 μl of PEI 40000 following the manufacturer’s protocol. Cells were transfected overnight for cell proliferation assay.

### Reverse transcription–PCR

Total RNA was extracted using the Steady Pure Quick RNA Extraction Kit (AG) according to the manufacturer’s instructions and used for first-strand cDNA synthesis using the Evo M-MLVRT Mix Kit (AG). The quantification of all gene transcripts was performed on a QuantStudioTM5 Real-Time PCR Instrument (Applied Biosystems Inc., Foster City, CA, USA) using the SYBR R Green Premix Pro Taq HS qPCR Kit (Rox Plus, Hunan, China) (AG), and RNA levels were normalized to those of 18S rRNA. 2^−ΔΔct^ was applied to analyze the data. Three parallel duplicate wells were designed for the experiment, and all samples were tested three times. Error bars represent the mean ± standard deviation (SD) from three independent experiments. The primer sequences used were as follows: 18S rRNA forward: 5′-GTAACCCGTTGAACCCCATT-3′, reverse: 5′-CCATCCAATCCGTAGTAGCG-3′; CEBPB, forward: 5′ TTTCGAAGTTGATGCAATCGG-3′, reverse: 5′-CGTAGGAACATCTTTAAGCGA-3′.

### Assessment of cell proliferation with cell counting kit-8 test

Cells were divided into the control group and the overexpression-CEBPB group. The cells were spread in 96-well plates with 3,000 cells per well, and then an appropriate amount of Cell Counting Kit-8 (CCK-8) reagent was added to detect the absorbance of each experimental group, so as to measure the proliferation activity at different times.

### Differential expression analysis

Differential expression analysis between metastatic SKCM samples with higher and lower CEBPB mRNA levels was performed by using the DESeq2 R package (31) based on the raw count data. For the single-cell RNA-seq, which provides fragments per kilobase of transcript per million mapped reads (FPKM) data for each cell, the limma R package (https://bioconductor.org/packages/release/bioc/html/limma.html) was applied for differential expression analysis between cells with CEBPB FPKM > 1 and those without CEBPB detected. Threshold of absolute log2(Fold Change) (|log2FC|) > 1 and false discovery rate (FDR) adjusted p-value<0.05 was used as the criteria for screening significantly differentially expressed genes (DEGs).

### Functional enrichment analysis

Significantly enriched Kyoto Encyclopedia of Genes and Genomes (KEGG) pathways were obtained by using the clusterProfiler R package (32) to the DEGs with the threshold of Benjamini–Hochberg (BH) adjusted p-value<0.05. In addition, Gene Set Enrichment Analysis (GSEA) was also adopted to identify hallmarks (gene signatures) that are associated with CEBPB expression in both bulk metastatic SKCM tissue and the macrophages in the TME by using GSEA software (33) with the threshold of nominal p-value<0.05.

### Construction of death risk signature

Univariate Cox regression analysis was first applied to the candidate genes, namely, CEBPB-regulated genes in macrophages, to identify prognostically significant genes based on the metastatic melanoma samples in the TCGA-SKCM cohort with the threshold of p-value<0.05. Least absolute shrinkage and selection operator (LASSO) Cox regression analysis by using the glmnet R package (https://cran.r-project.org/web/packages/glmnet/) was then performed to further prioritize genes from the significant ones in univariate Cox regression analysis for death risk signature construction. The signature was finally constructed as an equation for the estimation of death risk for each metastatic SKCM patient: risk score (RS) = $\sum_{i=1}^{n}Coe_{i}\times E_{i}$ , in which *Coe_i_* and *E_i_* represent the LASSO Cox coefficient and mRNA level of the *i*th gene, respectively.

### Survival analysis

Kaplan–Meier (KM) analysis by using the survival R package (https://CRAN.R-project.org/package=survival) was used for determining the significance of the difference in OS probability between metastatic SKCM patients with higher and lower CEBPB expression as well as with higher and lower RS. p-Value<0.05 was used as the significant threshold.

### Statistical analysis and visualization

A comparison of expression of CEBPB and antigen presentation-related genes between different patient groups was performed by using the Wilcoxon test with the threshold of p-value<0.05. Visualization of gene expression boxplot and volcano plot of differential expression analysis was performed through the ggplot2 R package (https://ggplot2-book.org/). The KM survival probability plot was visualized through the survminer R package (https://cran.r-project.org/web/packages/survminer/). The expression heatmap was visualized through the pheatmap R package (https://cran.rstudio.com/web/packages/pheatmap/). All the statistical analyses of this study were performed in R version 4.0.2 (https://www.r-project.org/).

## Results

### CEBPB is downregulated in metastatic skin cutaneous melanoma and associated with favorable prognosis

**Figure 1:**
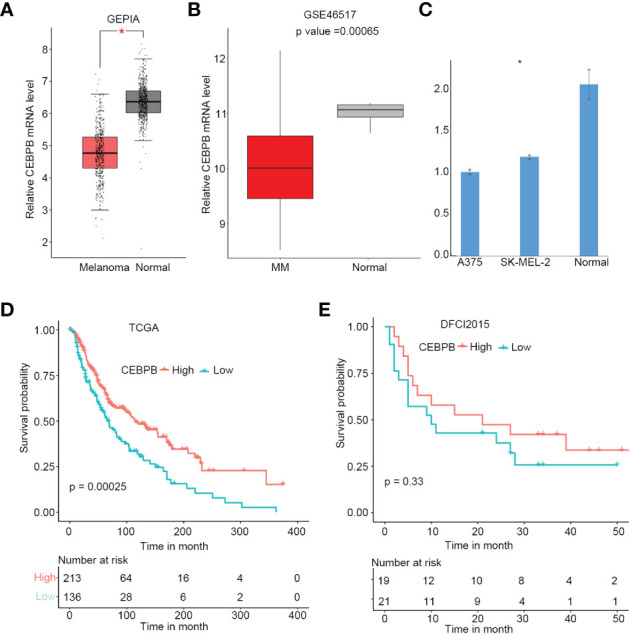
CEBPB is downregulated in metastatic SKCM and associated with superior prognosis. **(A)** Boxplot of relative CEBPB mRNA level in SKCM tumor samples from TCGA and normal skin samples from GTEx analyzed based on GEPIA. Asterisk indicates significant difference. **(B)** Boxplot of relative CEBPB mRNA level in metastatic SKCM tumor samples and normal skin samples from GSE46517. Exact p-value was provided. **(C)** Relative mRNA level of CEBPB in normal dermal fibroblast, A375, and SK-MEL-2 melanoma cell lines. **(D, E)** The KM survival probability curve of metastatic SKCM patients from TCGA-SKCM and DFCI2015 cohorts. The patients were grouped according to the best cut-point of CEBPB expression obtained through survminer R package. p-Value was determined by using log-rank test. SKCM, skin cutaneous melanoma; TCGA, The Cancer Genome Atlas; GTEx, Genotype-Tissue Expression; GEPIA, Gene Expression Profiling Interactive Analysis; KM, Kaplan–Meier.

To explore alterations of CEBPB expression in SKCM initiation, we first performed a comparison analysis through GEPIA online tool (http://gepia.cancer-pku.cn/), which includes SKCM tumor tissue samples from TCGA and normal tissue samples from GTEx. As a result, CEBPB was significantly downregulated in SKCM tumor tissues (**Figure 1A**), which was further confirmed by another independent cohort GSE46517, which included 73 metastatic SKCM samples and seven normal skin samples (primary and nevus samples were excluded from this study) (**Figure 1B**). To validate the downregulation of CEBPB in melanoma, we performed RT-PCR of CEBPB in dermal fibroblast from the normal skin of a keloid patient and A375 and SK-MEL-2 melanoma cells. As a result, the CEBPB mRNA level was significantly lower in A375 and SK-MEL-2 cells compared with that in dermal fibroblast (**Figure 1C**). To confirm the repression of CEBPB protein expression in SKCM tissue, we obtained the immunohistochemical staining results of CEBPB in three normal skin and three tumor tissues of malignant melanoma patients from the Human Protein Atlas (HPA; https://www.proteinatlas.org/). The staining intensity of CEBPB is obviously higher in normal skin samples than that in melanoma tumor tissues (**Figure S1**). Additionally, a high CEBPB mRNA level was proved to be associated with a favorable prognosis, namely, longer overall survival here, of metastatic SKCM in both the TCGA cohort (**Figure 1D**) and DFCI2015, another independent patient cohort (**Figure 1E**, not significant, which might be attributed to its relatively small sample size, but the survival probability difference between the two groups is obvious). These indicated that CEBPB could inhibit the initiation and progression of SKCM.

To explore if CEBPB has any influence on melanoma proliferation, we overexpressed CEBPB in both A375 and SK-MEL-2 cell lines and performed a CCK-8 assay. Strikingly, CEBPB overexpression (OE-CEBPB) almost had no influence on the proliferation ability of the two cell lines (**Figure S2**). We speculated that CEBPB might affect skin melanoma mainly by influencing other cell types in the tumor microenvironment rather than the malignant cell itself.

### CEBPB is associated with immune pathway activity in metastatic skin cutaneous melanoma

**Figure 2:**
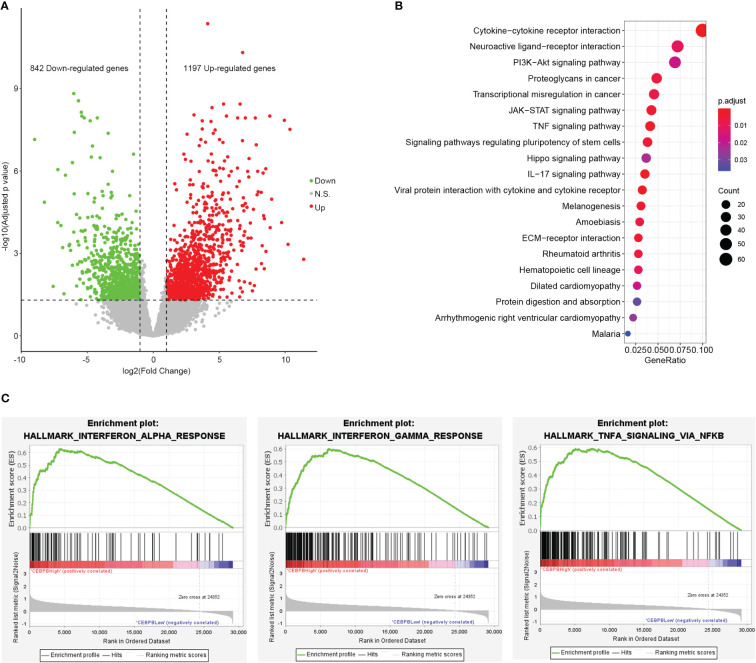
CEBPB is closely associated with immune response in metastatic SKCM tumor tissue. **(A)** Volcano plot of differential expression analysis between the 10 metastatic SKCM patients with the highest CEBPB expression (CEBPB_High) and the 10 with the lowest CEBPB expression (CEBPB_Low) from TCGA-SKCM cohort. Green, red, and gray dots represent significant downregulated, upregulated, and non-significant genes in CEBPB_High samples compared with CEBPB_Low samples. x-Axis and y-axis are log2FC and log10-based FDR-adjusted p-value, respectively. **(B)** The top 20 most significantly enriched KEGG pathways of the 2,039 DEGs in CEBPB_High samples. Dot size and color illustrate the number of genes included in the corresponding pathway and significance, respectively. Full list of significant KEGG pathways is provided in **Supplementary Table 1**. **(C)** Enrichment plot of three hallmark gene sets that are significantly enriched and specifically activated in the CEBPB_High samples compared with CEBPB_Low samples. The full list of significantly enriched gene sets in CEBPB_High samples is provided in **Supplementary Table 2**. SKCM, skin cutaneous melanoma; FDR, false discovery rate; KEGG, Kyoto Encyclopedia of Genes and Genomes; DEGs, differentially expressed genes.

CEBPB is a transcription factor that plays important roles in gene expression regulation. To identify genes regulated by CEBPB in metastatic SKCM tumor tissue, we selected the 10 metastatic SKCM tumor tissues that have the highest CEBPB expression (CEBPB_High) and the 10 with the lowest CEBPB expression (CEBPB_Low) from the TCGA cohort and conducted differential gene expression analysis between the two sample groups. As a result, a total of 2,039 DEGs, including 842 downregulated and 1,197 upregulated genes, were obtained in CEBPB_High compared with CEBPB_Low samples (**Figure 2A**). Functional enrichment analysis of those DEGs obtained many immune-related or TME regulation-related pathways, such as cytokine–cytokine interaction, JAK–STAT signaling pathway, and TNF signaling pathway (**Figure 2B**; **Supplementary Table 1**). GSEA indicated that immune response-related pathways were significantly activated in CEBPB_High metastatic SKCM samples (**Figure 2C**; **Supplementary Table 2**). These indicate that CEBPB participates in the immune activation in metastatic SKCM.

### CEBPB is mainly expressed in macrophages of metastatic skin cutaneous melanoma tumor tissue and is associated with immune activation

**Figure 3:**
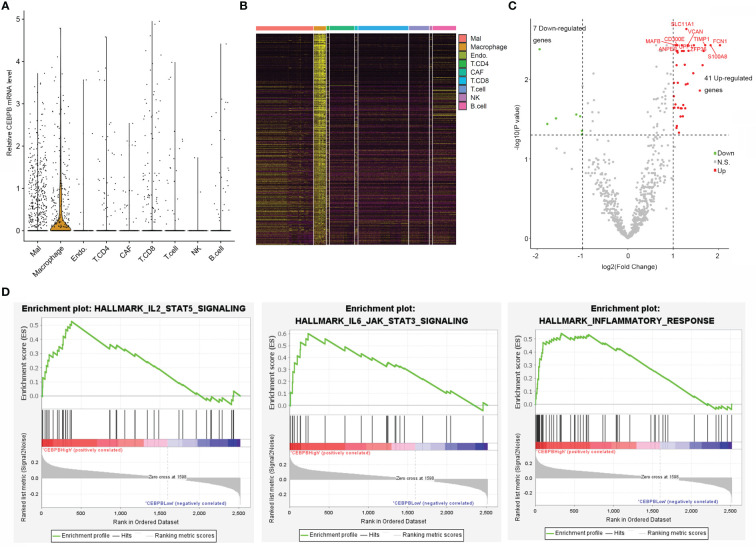
CEBPB is mainly expressed in macrophages of metastatic SKCM TME and associated with the immune activity of macrophages. **(A)** Distribution of CEBPB expression across different cell types in the metastatic SKCM TME. **(B)** Heatmap illustrating the expression of macrophage-specific markers across different cell types in the metastatic SKCM TME. **(C)** Volcano plot of differential expression analysis between macrophages with CEBPB FPKM > 1 and those macrophages without CEBPB detected. Only macrophage-specific genes were included in this analysis, and CEBPB itself was not included in this plot. Green, red, and gray dots represent genes that were significantly downregulated, upregulated, and non-significant in CEBPB FPKM > 1 macrophage compared with those without CEBPB detected. **(D)** Enrichment plot of three significantly enriched, specifically activated hallmark gene sets in CEBPB FPKM > 1 macrophage compared with macrophages without CEBPB detected. The full list of significantly enriched hallmark gene sets is provided in **Supplementary Table 3**. SKCM, skin cutaneous melanoma; TME, tumor microenvironment.

To explore if CEBPB was specifically expressed in some cell types in the TME of metastatic SKCM tumor tissue, we analyzed its mRNA level in a single cell RNA-seq dataset (GSE115978) of metastatic SKCM patients across multiple cell types. Strikingly, it was mainly expressed in macrophages but nearly depleted in other cell types (**Figure 3A**). CEBPB has been previously reported to be a master regulator in macrophages; we here proposed to explore genes regulated by CEBPB in macrophages of metastatic SKCM TME. We first identified macrophage-specific genes in the TME by comparing the gene expression in macrophages with other cell types with the threshold of |log2(Fold Change)| > 0.5 and FDR-adjusted p-value<0.05, which obtained a total of 2,516 genes (**Figure 3B**), and CEBPB was expectedly identified. Then we selected the macrophages that have no CEBPB mRNA detected and those with FPKM of CEBPB > 1 and performed differential expression analysis between those two macrophage groups. As a result, a total of 41 upregulated and seven downregulated genes in addition to CEBPB itself were identified in CEBPB_High macrophages compared with those without CEBPB detected (**Figure 3C**). Additionally, GSEA indicated that immune response-related pathways, such as inflammatory response and IL6–JAK–STAT3 signaling pathway, were significantly activated in CEBPB_High macrophages (**Figure 3D**; **Supplementary Table 3**), which was consistent with the bulk tumor tissue analysis. These indicated that CEBPB might manipulate the TME of metastatic SKCM by influencing the macrophage state.

### A macrophage-specific CEBPB-associated gene signature could robustly distinguish prognostically different metastatic skin cutaneous melanoma patients

**Figure 4:**
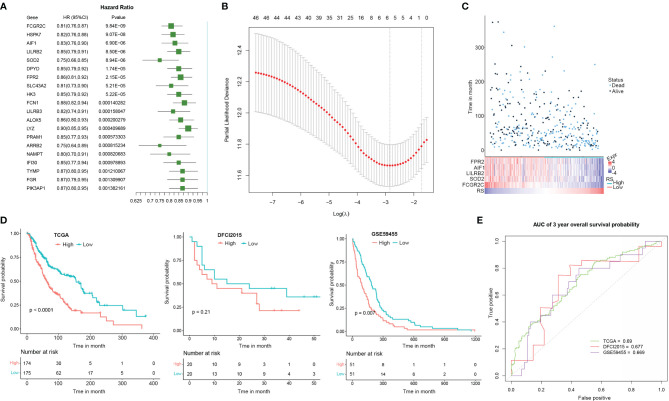
The signature based on the CEBPB-regulated genes in macrophages could robustly distinguish prognostically different metastatic SKCM patients. **(A)** Forest plot illustrating the hazard ratio (HR) and significance of the top 20 most significant CEBPB-regulated genes in macrophages in univariate Cox regression analysis of their association with overall survival probability of metastatic SKCM patients in TCGA-SKCM cohort. **(B)** LASSO Cox regression plot showing the optimal number of genes to be included in the death risk score (RS) signature. **(C)** RS and the overall survival time (month) as well as the survival status of each metastatic SKCM patient in TCGA-SKCM were altogether provided as a dot plot along with a heatmap. Each dot represents a patient, with dot color indicating the survival status, and the corresponding RS could be obtained through the last row of the heatmap. The relative expression of genes included in the signature was also illustrated in the heatmap. **(D)** KM survival probability curve of metastatic SKCM patients in TCGA-SKCM (left panel), DFCI2015 (middle panel), and GSE59455 (right panel) cohorts. The patients were grouped by the median RS value. p-Value was determined by using log-rank test. **(E)** Survival ROC curve for estimating the reliability of the RS in predicting the 3-year overall survival probability. The curve color represents the dataset used as indicated in the plot. SKCM, skin cutaneous melanoma; LASSO, least absolute shrinkage and selection operator; KM, Kaplan–Meier; ROC, receiver operating characteristic.

As TME state is closely associated with the prognosis of multiple cancers, we here proposed to explore if the CEBPB-associated genes could predict the prognosis of metastatic SKCM patients. We performed a univariate Cox regression analysis of the 48 DEGs in macrophages that were regulated by CEBPB along with CEBPB itself for their association with OS probability of metastatic SKCM patients from TCGA, which identified 36 prognostic-related genes. The top 20 most significant genes along with their hazard ratio and p-value were provided as a forest plot in **Figure 4A**. To further prioritize genes for prognostic signature construction, we applied the LASSO Cox regression method to the 36 prognostically significant genes, which finally retained five genes (**Figure 4B**), including FPR2, AIF1, LILRB2, SOD2, and FCGR2C, and the death RS of metastatic SKCM patients could be calculated by the sum of multiply–accumulate of each gene’s relative expression, namely, log2-based raw sequencing reads, and its regression coefficient: RS = −0.0194 × FPR2 − 0.0202 × AIF1 − 0.0254 × LILRB2 − 0.0387 × SOD2 − 0.1226 × FCGR2C. We calculated the RS for the metastatic SKCM patients in TCGA cohort and found that patients with high RS had relatively short OS than those with low RS (**Figure 4C**). Additionally, KM analysis also illustrated the association between high RS and inferior OS of metastatic SKCM patients in TCGA cohort as well as another two independent validation metastatic SKCM patient cohorts (**Figure 4D**). Receiver operating characteristic (ROC) curve analysis for the prediction ability of RS for 3-year OS probability of metastatic SKCM patients obtained the area under the curve (AUC) of TCGA, DFCI2015, and GSE59455 as 0.690, 0.677, and 0.669, respectively (**Figure 4E**), which indicated the good performance of RS in prognosis prediction.

### CEBPB-associated risk score is related to tumor immune landscape in metastatic skin cutaneous melanoma

**Figure 5:**
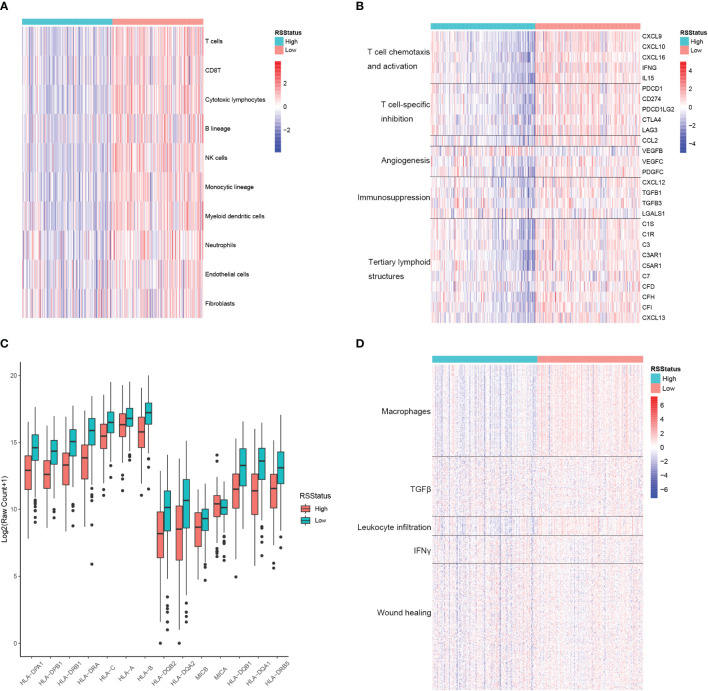
The signature based on the CEBPB-regulated genes in macrophages is closely associated with immune activity in metastatic SKCM TME. **(A)** Relative abundance of lymphocytes, myeloid cells, and stromal cells in metastatic SKCM patients’ tumor samples of the TCGA-SKCM cohort stratified by the median RS value. **(B)** Relative expression of genes of several immunologically suppressive and active signatures in metastatic SKCM patients’ tumor samples of the TCGA-SKCM cohort stratified by the median RS value. **(C)** Relative expression of antigen presentation-related genes in metastatic SKCM patients’ tumor samples of the TCGA-SKCM cohort with higher and lower RS stratified by the median RS value. Asterisk indicates a significant difference, namely, p-value< 0.05. n.s. represents non-significance. **(D)** Relative expression of genes used for defining immune cluster in the study of Shmulevich et al. (34) in the metastatic SKCM patients’ tumor tissues of the TCGA-SKCM cohort stratified by the median RS value. RSStatus, risk score status based on the median RS value; SKCM, skin cutaneous melanoma; TME, tumor microenvironment.

RS could robustly separate prognostically different metastatic SKCM patients, and the TME plays fundamental roles in cancer initiation, progression, and drug resistance, so we proposed to explore the potential association between RS and TME landscape in metastatic SKCM tissues. First, the relative infiltration level of lymphocyte, myeloid cell, and the stromal cell was determined by the MCPCounter algorithm and compared between metastatic SKCM patients of the TCGA cohort stratified by their median RS. As a result, T cells, CD8 T cells, cytotoxic lymphocytes, NK cells, and monocytic lineage cells were among the most differential cell types, which had significantly high infiltration levels in SKCM patients with low RS. In contrast, neutrophils, endothelial cells, and fibroblasts only showed a slight difference between the two patient groups (**Figure 5A**). We then comprehensively collected genes closely related to TME modulation, including cytokines and chemokines associated with immune activity, namely, T-cell chemotaxis and activation and tertiary lymphoid structures here, and those associated with immune suppressive, including T cell-specific suppression, angiogenesis, and immunosuppression, and we compared their expressions between metastatic SKCM patients with high and low RS. Expectedly, mRNA levels of all the genes related to T-cell chemotaxis and activation as well as tertiary lymphoid structures (TLSs) were significantly higher in metastatic SKCM patients with low RS than those with high RS. Strikingly, most of the genes related to immune suppressive also showed higher expression in RS_low patients except angiogenesis-related VEGFB and immunosuppression-related LGALS1, which were significantly higher in patients with higher RS (**Figure 5B**). This was partially controversial, for lower RS was associated with superior OS (**Figure 4**). We speculated that the immune active environment might stimulate the response of immunosuppression signals of multiple origins, such as high expression of immune checkpoint ligand gene in malignant cells and chemokine cascade secreted from tumor-associated macrophage (TAM) for regulatory T-cell recruitment. Antigen presentation (AP) is a core process for the recognition and the following cytotoxicity of T cells for malignant cells. Hence, we collected a comprehensive list of AP-related genes and compared their mRNA levels in metastatic SKCM patients from the TCGA cohort stratified by the median RS. As a result, 13 of the 14 genes showed significantly higher expressions in patients with low RS (RS_low) than in those with high RS (RS_high) as shown in **Figure 5C**, indicating that the RS_low patients had a more intact AP system, which might underlie the better OS of those patients. Additionally, Shmulevich et al. grouped cancer patients into five immune clusters according to gene expression signatures associated with immune activity (34). To explore the relationship between RS and the immune cluster, we collected the genes used for defining each immune cluster and compared their mRNA levels between RS_low and RS_high metastatic SKCM patients. As a result, genes enriched in the macrophage and leukocyte infiltration cluster showed obviously higher expressions in RS_low patients than RS_high patients, whereas differences in expressions of TGFβ, IFNγ, and wound healing-related genes were nearly indistinguishable between the two patient groups (**Figure 5D**). These indicated that low RS is associated with high immune activity and might have predictive value for immunotherapy response.

## Discussion

Metastatic SKCM is a common malignancy worldwide that accounts for low morbidity but very high mortality in all skin cancers (35). High exposure to UV irradiation represents the most common pathogenesis, which induces a high accumulation of mutation and in turn causes aggressive lymphocytic infiltration (36). Systemic treatment, including radiotherapy, chemotherapy, and the recently developed immunotherapy, are the most adopted therapeutics, and they provide some clinical benefits to metastatic SKCM, but heterogeneous clinical outcomes stand in the way of widespread adoption (37, 38). Therefore, it is urgently necessary to identify biomarkers or signatures that may immunologically and prognostically distinguish different patients.

In this study, we reported the associations between CEBPB and prognosis as well as the TME landscape of metastatic SKCM patients, and a genetic signature associated with CEBPB was developed for immune activity and stratification of OS probabilities. The results showed that high CEBPB expression is associated with a favorable prognosis of metastatic SKCM and activated immune response-related pathways in bulk tumor tissue as well as pure macrophages. It has been extensively reported that CEBPB participates in immune regulation (16, 39), and CEBPB contributes to immune activation in many scenarios and relates to the occurrence of many autoimmune diseases (40, 41). Particularly, it represents a master regulator for macrophage identity determination (21). Indeed, CEBPB has proven to be nearly specifically expressed in the macrophages of metastatic SKCM tissues and regulates several immune response-related pathways in both macrophage and bulk tumor tissue in this study. Macrophages exhibit controversial roles at different tumor stages. For example, macrophages could cause tumor regression by directly killing malignant cells or promoting the presentation of tumor cells to T cells. In contrast, macrophages might transform into TAMs and promote tumorigenesis, progression, metastasis, and drug resistance when type 2 T helper cell dominates the TME contexture (12). Above all, we speculated that CEBPB could prompt the polarization of macrophages to the immunologically stimulative subtype in metastatic SKCM TME and in turn inhibit tumor progression. In addition, the CEBPB mRNA level was significantly lower in melanoma cells than in normal dermal fibroblasts. In contrast, CEBPB overexpression almost had no influence on the proliferation ability of melanoma cells. CEBPB therefore might affect the initiation and progression of melanoma through the modulation of the tumor microenvironment rather than the malignant cell itself. In contrast, correlations between the change in malignant cells caused by CEBPB inhibition and tumor microenvironment perturbation should still be further studied.

For the important roles of macrophages in the determination of a relationship between the TME and tumor progression, we specially screened the macrophage-specific genes that were regulated by CEBPB in the metastatic SKCM and constructed a prognostic signature by using several CEBPB-regulated genes. The robustness of the gene signature in prognosis stratification was confidently validated with the assistance of three independent patient cohorts. In addition, we found obvious differences in the TME landscape between the signature-derived RS in high and low patients. Generally, the RS_low metastatic SKCM patients illustrated a more active immune environment than RS_high patients with respect to T-cell infiltration and state as well as antigen presentation. Unexpectedly, certain genes related to immunosuppression and angiogenesis also showed higher mRNA levels in the RS_low patient group than in the RS_high group. The TME is a complex contexture in which immunologically active and suppressive signals coexist and transition from high tumor immunity in the initial tumor to low tumor immunity in the late-stage tumor, which could occur in tumor progression (42, 43). In quantitative terms, the relative increment in the expression of immunologically active-related genes in RS_low samples compared to RS_high samples is much higher than that of immunologically suppressive-related genes, which might indicate that the RS could discriminate between patients with dominant immunoactive TME versus those with dominant immunosuppressants.

Immunotherapy is a recently developed therapeutic that aims to modulate the TME, and significant prognostic improvement has been achieved. However, the efficacy of immunotherapy is highly heterogeneous across different cancers and among individuals of the same cancer with differences in their immune environment (44). Some biomarkers, such as tumor mutation burden, neoantigen load, and lymphocytic infiltration, have been proposed to be associated with immunotherapy responses (45, 46). However, there is evidence that the predictive values are limited in numerous investigations. In this study, the RS is closely associated with TME state: the lower the RS, the more active the immunity. In addition, the DFCI2015 patient cohort is comprised of immunotherapy-experienced metastatic SKCM patients, and the RS could robustly discriminate against the different prognostic patients. These indicate the potential predictive value of the RS in the immunotherapy response of metastatic SKCM patients.

This study also has several limitations. First of all, the conclusion about the influence of CEBPB on the tumor microenvironment of melanoma patients was mainly based on the prediction of the immune cell infiltration ratio in tumor tissues. An animal model with an intact immune system along with CEBPB perturbation would be more precise for exploring associations between CEBPB and the tumor microenvironment. Another limitation of this study was the lack of normal melanocytes for validation of CEBPB mRNA level, which was superseded by dermal fibroblast, although the CEBPB expression was indeed very low in A375 and SK-MEL-2 melanoma cells. These efforts will continue into the future.

## Conclusion

In conclusion, we validated the positive role of CEBPB in metastatic SKCM patients and its function in enabling the immune response pathway in both bulk SKCM tissue and macrophages of SKCM TME for the first time. Additionally, a signature based on genes regulated by CEBPB in macrophages was also constructed, which could robustly distinguish prognostically and immunologically different metastatic SKCM patients. It might be a potentially useful marker for immunotherapy response.

## Data availability statement

The original contributions presented in the study are included in the article/**Supplementary Material**. Further inquiries can be directed to the corresponding authors.

## Author contributions

KX performed data curation and formal analysis. LG performed formal analysis. WZ performed the investigation. XL performed the writing – review and editing. JY and YX performed experiments and writing of the revised manuscript. All authors contributed to the article and approved the submitted version.

## Funding

This work was supported by the research start-up funds of Hangzhou Normal University (No. 4125C50221204039) and National Natural Science Foundation of China (No. 82203471).

## Conflict of interest

The authors declare that the research was conducted in the absence of any commercial or financial relationships that could be construed as a potential conflict of interest.

## Publisher’s note

All claims expressed in this article are solely those of the authors and do not necessarily represent those of their affiliated organizations, or those of the publisher, the editors and the reviewers. Any product that may be evaluated in this article, or claim that may be made by its manufacturer, is not guaranteed or endorsed by the publisher.

## Supplementary material

Supplementary Figure 1 Immunohistochemical staining of CEBPB in normal skin samples and melanoma tumor tissues from HPA database. Patient ID in HPA were provided for each staining image.

Supplementary Figure 2 Cell proliferation ability detected by CCK-8 assay for control and OE-CEBPB A375 (A) and SK-MEL-2 (B) cell lines.

## References

[1] Shenenberger DW. Cutaneous malignant melanoma: a primary care perspective. Am Fam Phys (2012) 85(2):161–8. 22335216

[2] Berk-Krauss J Stein JA Weber J Polsky D Geller AC. New systematic therapies and trends in cutaneous melanoma deaths among US whites, 1986-2016. Am J Public Health (2020) 110(5):731–3. doi: 10.2105/AJPH.2020.305567 PMC7144422 32191523

[3] Lugowska I Teterycz P Rutkowski P. Immunotherapy of melanoma. Contemp Oncol (Pozn) (2018) 22(1A):61–7. doi: 10.5114/wo.2018.73889 PMC5885078 29628796

[4] Kee D McArthur G. Targeted therapies for cutaneous melanoma. Hematol Oncol Clin North Am (2014) 28(3):491–505. doi: 10.1016/j.hoc.2014.02.003 24880943

[5] Miller KD Nogueira L Mariotto AB Rowland JH Yabroff KR Alfano CM. Cancer treatment and survivorship statistics, 2019. CA Cancer J Clin (2019) 69(5):363–85. doi: 10.3322/caac.21565 31184787

[6] Bhatia S Tykodi SS Thompson JA. Treatment of metastatic melanoma: an overview. Oncol (Williston Park) (2009) 23(6):488–96. PMC2737459 19544689

[7] Paijens ST Vledder A de Bruyn M Nijman HS. Tumor infiltrating lymphocytes (TIL) therapy in metastatic melanoma: boosting of neoantigen-specific T cell reactivity and long-term follow-up. J Immunother Cancer (2020) 8(2):1–11. doi: 10.1136/jitc-2020-000848 PMC7406109 32753545

[8] Tao T Liu Y Zhang J Huang L Tao Y. Dynamic observation: Immune-privileged microenvironment limited the effectiveness of immunotherapy in an intraocular metastasis mouse model. Ophthal Res (2022) 53. doi: 10.1159/000524485 35398850

[9] Tschernia NP Gulley JL. Tumor in the crossfire: Inhibiting TGF-beta to enhance cancer immunotherapy. BioDrugs (2022) 36(2):153–80. doi: 10.1007/s40259-022-00521-1 PMC8986721 35353346

[10] Harlin H Meng Y Peterson AC Zha Y Tretiakova M Slingluff C. Chemokine expression in melanoma metastases associated with CD8+ T-cell recruitment. Cancer Res (2009) 69(7):3077–85. doi: 10.1158/0008-5472.CAN-08-2281 PMC3886718 19293190

[11] Jiang Y Li Y Zhu B. T-Cell exhaustion in the tumor microenvironment. Cell Death Dis (2015) 6:e1792. doi: 10.1038/cddis.2015.162 26086965 PMC4669840

[12] Lopez-Yrigoyen M Cassetta L Pollard JW. Macrophage targeting in cancer. Ann N Y Acad Sci (2021) 1499(1):18–41. doi: 10.1111/nyas.14377 32445205

[13] Lefler JE Seward C Ostrowski MC. PTEN in cancer associated fibroblasts. Adv Cancer Res (2022) 154:203–26. doi: 10.1016/bs.acr.2022.01.002 35459470

[14] Zou Y Zheng S Deng X Yang A Xie X Tang H. The role of circular RNA CDR1as/ciRS-7 in regulating tumor microenvironment: A pan-cancer analysis. Biomolecules (2019) 9(9):1–13. doi: 10.3390/biom9090429 PMC6770779 31480381

[15] Sterken BA Ackermann T Muller C Zuidhof HR Kortman G Hernandez-Segura A. C/EBPbeta isoform-specific regulation of migration and invasion in triple-negative breast cancer cells. NPJ Breast Cancer (2022) 8(1):11. doi: 10.1038/s41523-021-00372-z 35042889 PMC8766495

[16] Xu C Xu J Lu L Tian W Ma J Wu M. Identification of key genes and novel immune infiltration-associated biomarkers of sepsis. Innate Immun (2020) 26(8):666–82. doi: 10.1177/1753425920966380 PMC7787554 33100122

[17] McPeak MB Youssef D Williams DA Pritchett CL Yao ZQ McCall CE. Frontline science: Myeloid cell-specific deletion of cebpb decreases sepsis-induced immunosuppression in mice. J Leukoc Biol (2017) 102(2):191–200. doi: 10.1189/jlb.4HI1216-537R 28476751 PMC5505744

[18] Qie C Jiang J Liu W Hu X Chen W Xie X. Single-cell RNA-seq reveals the transcriptional landscape and heterogeneity of skin macrophages in vsir(-/-) murine psoriasis. Theranostics (2020) 10(23):10483–97. doi: 10.7150/thno.45614 PMC7482809 32929361

[19] Satoh T Nakagawa K Sugihara F Kuwahara R Ashihara M Yamane F. Identification of an atypical monocyte and committed progenitor involved in fibrosis. Nature (2017) 541(7635):96–101. doi: 10.1038/nature20611 28002407

[20] Vittal R Mickler EA Fisher AJ Zhang C Rothhaar K Gu H. Type V collagen induced tolerance suppresses collagen deposition, TGF-beta and associated transcripts in pulmonary fibrosis. PloS One (2013) 8(10):e76451. doi: 10.1371/journal.pone.0076451 24204629 PMC3804565

[21] Kuznetsova T Prange KHM Glass CK Winther MPJ. Transcriptional and epigenetic regulation of macrophages in atherosclerosis. Nat Rev Cardiol (2020) 17(4):216–28. doi: 10.1038/s41569-019-0265-3 PMC7770754 31578516

[22] Shao K Pu W Zhang J Guo S Qian F Glurich I. DNA Hypermethylation contributes to colorectal cancer metastasis by regulating the binding of CEBPB and TFCP2 to the CPEB1 promoter. Clin Epigenet (2021) 13(1):89. doi: 10.1186/s13148-021-01071-z PMC8063327 33892791

[23] Wang S Xia D Wang X Cao H Wu C Sun Z. C/EBPbeta regulates the JAK/STAT signaling pathway in triple-negative breast cancer. FEBS Open Bio (2021) 11(4):1250–8. doi: 10.1002/2211-5463.13138 PMC8016132 33660927

[24] Swoboda A Soukup R Eckel O Kinslechner K Wingelhofer B Schörghofer D. STAT3 promotes melanoma metastasis by CEBP-induced repression of the MITF pathway. Oncogene (2021) 40(6):1091–105. doi: 10.1038/s41388-020-01584-6 PMC7116782 33323974

[25] Vidarsdottir L Fernandes RV Zachariadis V Das I Edsbäcker E Sigvaldadottir I. Silencing of CEBPB-AS1 modulates CEBPB expression and resensitizes BRAF-inhibitor resistant melanoma cells to vemurafenib. Melanoma Res (2020) 30(5):443–54. doi: 10.1097/CMR.0000000000000675 PMC7469874 32467529

[26] Van Allen EM Miao D Schilling B Shukla SA Blank C Zimmer L. Genomic correlates of response to CTLA-4 blockade in metastatic melanoma. Science (2015) 350(6257):207–11. doi: 10.1126/science.aad0095 PMC5054517 26359337

[27] Budden T Davey RJ Vilain RE Ashton KA Braye SG Beveridge NJ. Repair of UVB-induced DNA damage is reduced in melanoma due to low XPC and global genome repair. Oncotarget (2016) 7(38):60940–53. doi: 10.18632/oncotarget.10902 PMC5308628 27487145

[28] Kabbarah O Nogueira C Feng B Nazarian RM Bosenberg M Wu M. Integrative genome comparison of primary and metastatic melanomas. PloS One (2010) 5(5):e10770. doi: 10.1371/journal.pone.0010770 20520718 PMC2875381

[29] Jerby-Arnon L Shah P Cuoco MS Rodman C Su M-J Melms JC. A cancer cell program promotes T cell exclusion and resistance to checkpoint blockade. Cell (2018) 175(4):984–997.e24. doi: 10.1016/j.cell.2018.09.006 30388455 PMC6410377

[30] Xie K Peng Y Zhong W Liu X. KMT2C is a Potential Biomarker of Anti-PD-1 Treatment Response in Metastatic Melanoma. Front Biosci (Landmark Ed) (2022) 27(3):103. doi: 10.31083/j.fbl2703103 35345335

[31] Love MI Huber W Anders S. Moderated estimation of fold change and dispersion for RNA-seq data with DESeq2. Genome Biol (2014) 15(12):550. doi: 10.1186/s13059-014-0550-8 25516281 PMC4302049

[32] Yu G Wang LG Han Y He Q-Y. clusterProfiler: an r package for comparing biological themes among gene clusters. OMICS (2012) 16(5):284–7. doi: 10.1089/omi.2011.0118 PMC3339379 22455463

[33] Subramanian A Tamayo P Mootha VK Mukherjee S Ebert BL Gillette MA. Gene set enrichment analysis: a knowledge-based approach for interpreting genome-wide expression profiles. Proc Natl Acad Sci U.S.A. (2005) 102(43):15545–50. doi: 10.1073/pnas.0506580102 PMC1239896 16199517

[34] Thorsson V Gibbs DL Brown SD Wolf D Bortone DS Ou Yang T-H. The immune landscape of cancer. Immunity (2018) 48(4):812–830.e14. doi: 10.1016/j.immuni.2018.03.023 29628290 PMC5982584

[35] Strickley JD Jenson AB Jung JY. Cutaneous metastasis. Hematol Oncol Clin North Am (2019) 33(1):173–97. doi: 10.1016/j.hoc.2018.08.008 30497674

[36] Wang J Perry CJ Meeth K Thakral D Damsky W Micevic G. UV-Induced somatic mutations elicit a functional T cell response in the YUMMER1.7 mouse melanoma model. Pigment Cell Melanoma Res (2017) 30(4):428–35. doi: 10.1111/pcmr.12591 PMC5820096 28379630

[37] Ralli M Botticelli A Visconti IC Angeletti D Fiore M Marchetti P. Immunotherapy in the treatment of metastatic melanoma: Current knowledge and future directions. J Immunol Res (2020), 9235638. doi: 10.1155/2020/9235638 32671117 PMC7338969

[38] Lee AY Brady MS. Neoadjuvant immunotherapy for melanoma. J Surg Oncol (2021) 123(3):782–8. doi: 10.1002/jso.26229 PMC8366312 33002195

[39] Li W Tanikawa T Kryczek I Xia H Li G Wu K. Aerobic glycolysis controls myeloid-derived suppressor cells and tumor immunity via a specific CEBPB isoform in triple-negative breast cancer. Cell Metab (2018) 28(1):87–103.e6. doi: 10.1016/j.cmet.2018.04.022 29805099 PMC6238219

[40] Chen J-Y Wang C-M Ma C-C Chow Y-H Luo S-F. The -844C/T polymorphism in the fas ligand promoter associates with Taiwanese SLE. Genes Immun (2005) 6(2):123–8. doi: 10.1038/sj.gene.6364158 15674374

[41] Lin Y Huang M Wang S You X Zhang L Chen Y. PAQR11 modulates monocyte-to-macrophage differentiation and pathogenesis of rheumatoid arthritis. Immunology (2021) 163(1):60–73. doi: 10.1111/imm.13303 33421113 PMC8044334

[42] Duan Q Zhang H Zheng J Zhang L. Turning cold into hot: Firing up the tumor microenvironment. Trends Cancer (2020) 6(7):605–18. doi: 10.1016/j.trecan.2020.02.022 32610070

[43] Weber CE Kuo PC. The tumor microenvironment. Surg Oncol (2012) 21(3):172–7. doi: 10.1016/j.suronc.2011.09.001 21963199

[44] De Cicco P Ercolano G Ianaro A. The new era of cancer immunotherapy: Targeting myeloid-derived suppressor cells to overcome immune evasion. Front Immunol (2020) 11:1680. doi: 10.3389/fimmu.2020.01680 32849585 PMC7406792

[45] Schumacher TN Schreiber RD. Neoantigens in cancer immunotherapy. Science (2015) 348(6230):69–74. doi: 10.1126/science.aaa4971 25838375

[46] Paijens ST Vledder A de Bruyn M Nijman HW. Tumor-infiltrating lymphocytes in the immunotherapy era. Cell Mol Immunol (2021) 18(4):842–59. doi: 10.1038/s41423-020-00565-9 PMC8115290 33139907

