# Supplementary figures and images for: CEBPB is associated with active tumor immune environment and favorable prognosis of metastatic skin cutaneous melanoma
Source: Front Immunol. 2022 Oct 24;13:991797. doi: 10.3389/fimmu.2022.991797 (PMC9637891; doi:10.3389/fimmu.2022.991797)

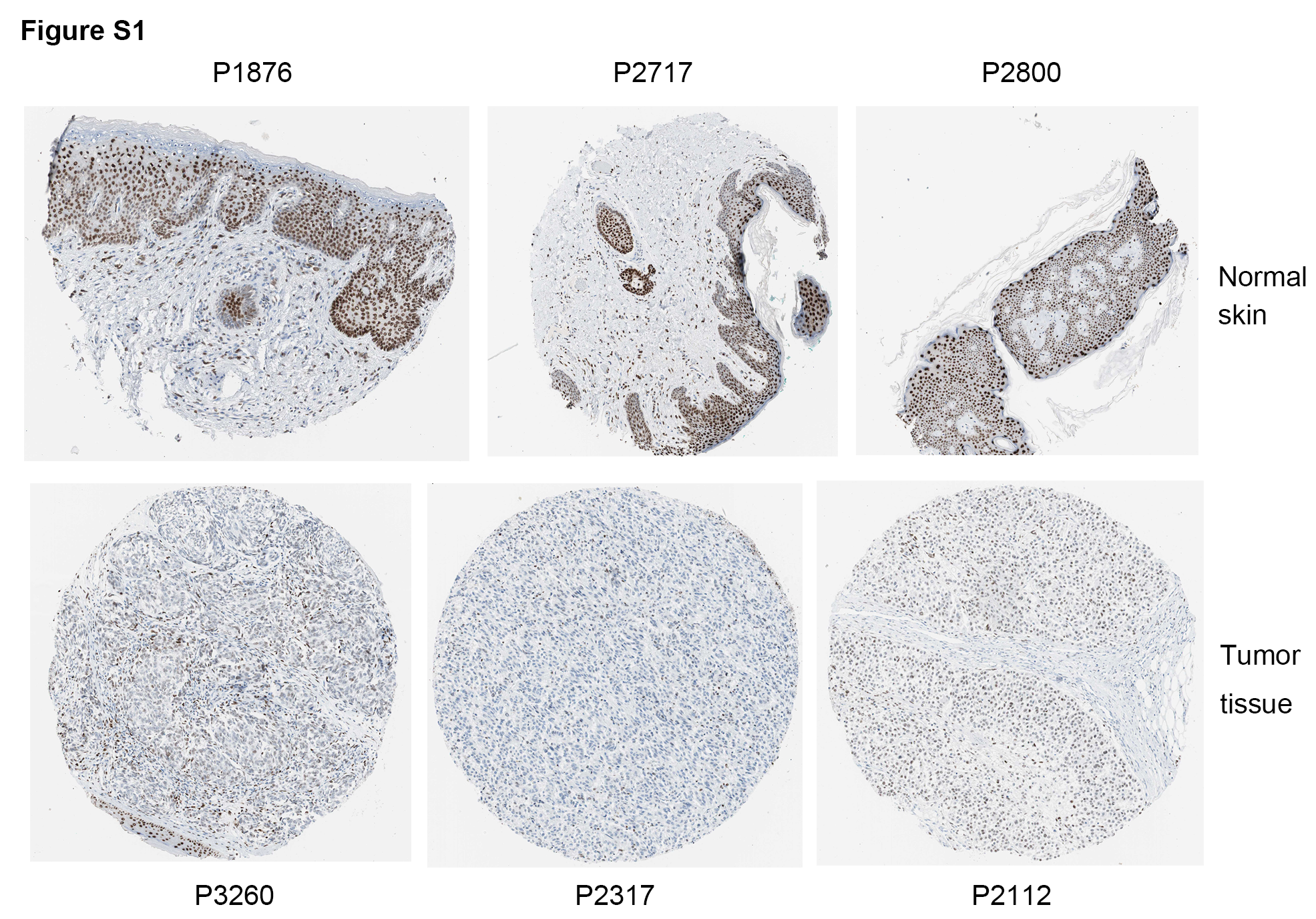

Supplement: Supplementary Figure 1 — Immunohistochemical staining of CEBPB in normal skin samples and melanoma tumor tissues from HPA database. Patient ID in HPA were provided for each staining image. [file Image_1.tif]

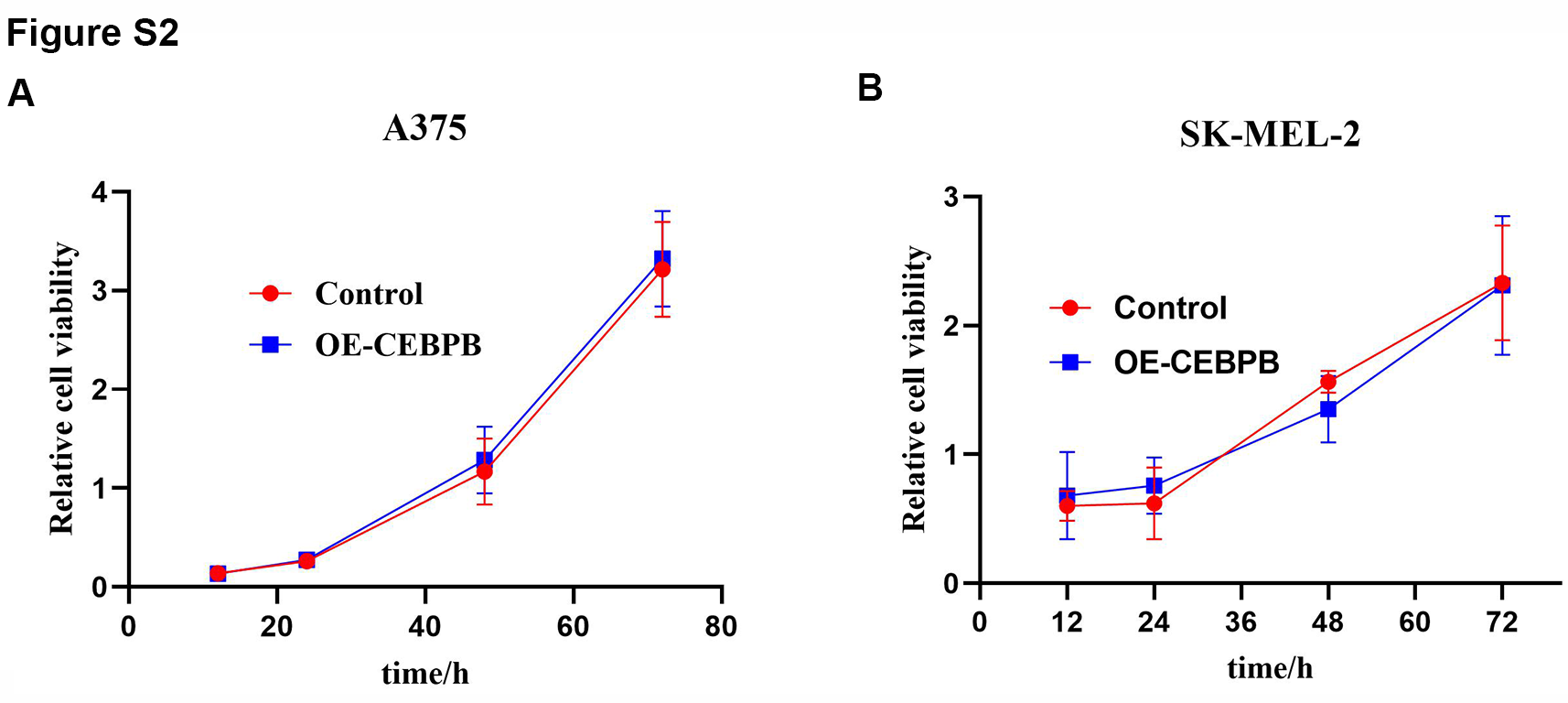

Supplement: Supplementary Figure 2 — Cell proliferation ability detected by CCK-8 assay for control and OE-CEBPB A375 (A) and SK-MEL-2 (B) cell lines. [file Image_2.tif]
